# Next-generation sequencing yields the complete mitogenome of the blue-banded sea-snake (Squamata: Elapidae)

**DOI:** 10.1080/23802359.2019.1630334

**Published:** 2019-07-12

**Authors:** Qingbo Qiu, Qize Liu, Yifan Zhao, Yu Du, Chixian Lin, Xiang Ji

**Affiliations:** Hainan Key Laboratory of Herpetological Research, College of Fisheries and Life Science, Hainan Tropical Ocean University, Sanya, China

**Keywords:** Complete mitochondrial genome, phylogeny, Elapidae, *Hydrophis cyanocinctus*

## Abstract

We report the complete mitogenome of *Hydrophis cyanocinctus*, which is 17,750 bp in size and includes 13 protein-coding (PCGs), 2 rRNA genes, 22 tRNA genes, and 2 control regions. PCGs, with 13 genes, is 11,427 bp in length. All PCGs use ATN as the typical start codon except COX1 with GTG; the TAG was found as the stop codon in ND1 and ND2, the AGA was found as the stop codon in COI and ND6, the TAA was found as the stop codon in ATP8, ATP6, ND4L, ND5 and Cytb; while other 3 PCGs stop with a single T. Phylogeny reconstructed using the Bayesian inference (BI) method with 13 PCGs indicates the presence of *H. cyanocinctus* at the root of Laticaudinae.

Blue-banded sea-snake (*Hydrophis cyanocinctus*) is a viviparous elapid species and inhabits in shallow coastal waters around western Pacific Ocean and Indian Ocean (Zhao and Adler 1993). Mitogenomic data utilized to reconstrue phylogeny indicate the evolutionary history. However, it is available for only 9 species (including 4, 3 and 2 species in Elapinae, Laticaudinae and Bungarinae, respectively) of the more than 377 species in Elapid (Uetz et al. 2019). In order to obtain more basic genetic information on this group of snakes, we determined the complete mitogenome of *H. cyanocinctus*.

The sample (HKHR302) was collected from Hainan, China, and stored at ‒80 °C in our laboratory at Hainan Tropical Ocean University. Total genomic DNA was isolated using EasyPure@ Genomic DNA Kit according to the manufacturer’s instructions (TransGen Biotech Co, Beijing, China). The mitogenomes of *H. cyanocinctus* were sequenced by next-generation sequencing (Illumina HiSeq X Ten; Novogene Bioinformatics Technology Co. Ltd., Nanjing, China) for PE 2 × 150 BP sequencing. Raw sequence data (32.52 Gb) were deposited into Genome Sequence Archive (GSA) database (http://bigd.big.ac.cn/gsa/) with the accession no. CRA001561, and clean data without sequencing adapters were *de novo* assembled using the NOVOPlasty 2.7.2 (Dierckxsens et al. 2017).

The complete and circular mitochondrial genome is 17,750 bp in size, it has been deposited in the Genome Warehouse (BIG Data Center Members 2019, accession: GWHAATA00000000) and genbank (accession: MK953550) with an AT bias of 60.3%, and includes 13 protein-coding genes(PCGs), 22 transfer RNA genes (tRNA), 2 ribosomal RNA genes (12S rRNA and 16S rRNA), and 2 non-coding regions (CR or D-loop). There are ND6 subunit gene and eight tRNAs on the L-strand, and the other 28 genes in *H. cyanocinctus* on the H-strand. Thirteen PCGs are 11,427 bp and start with typical ATN codons except COX1 with GTG; the TAG was found as the stop codon in ND1 and ND2, the AGA was found as the stop codon in COI and ND6, the TAA was found as the stop codon in ATP8, ATP6, ND4L, ND5, and Cytb; while 3 PCGs (ATP6, COIII and ND3) stop with a single T. The 22 tRNA genes varied in size from 57 to 73 bp.

Using *Bothrops pubescens* and *Gloydius strauchi* as outgroups, we used the Mrbayes 3.1.2 to reconstruct the phylogeny of 10 Elapid species including *H. cyanocinctus* based on 13 PCGs. The resultant BI tree distinctly indicated that *H. cyanocinctus* belongs to a monophyletic group including Elapinae, Laticaudinae and Bungarinae, and is located at the root of the Laticaudinae (Figure 1).

**Figure 1.**
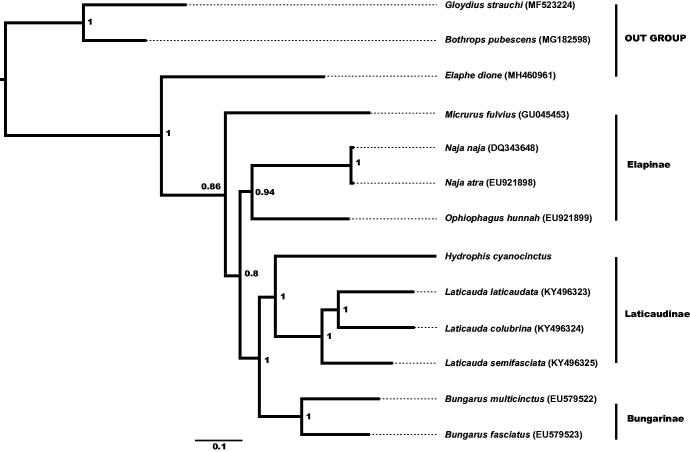
Bayesian inference (BI) phylogenetic tree inferred from the nucleotide sequence data of mitogenomic 13 PCGs.
